# Self-Reported Sleep and Exercise Patterns in Patients with Schizophrenia: a Cross-Sectional Comparative Study

**DOI:** 10.1007/s12529-019-09830-2

**Published:** 2019-12-17

**Authors:** Nancy Kiwan, Ziyad Mahfoud, Suhaila Ghuloum, Rifka Chamali, Arij Yehya, Samer Hammoudeh, Yahya Hani, Iman Amro, Hassen Al-Amin

**Affiliations:** 1 Department of Research, Weill Cornell Medicine-Qatar, Doha, Qatar; 2 Department of Health Policy and Research, Weill Cornell Medicine-Qatar, Doha, Qatar; 3 Department of Healthcare Policy and Research, Weill Cornell Medicine, New York, NY USA; 4 Psychiatry Hospital, Hamad Medical Corporation, Doha, Qatar; 5 Department of Psychiatry, Weill Cornell Medicine-Qatar, Education City, P.O. Box 24144, Doha, Qatar

**Keywords:** Schizophrenia, Sleep duration and quality, Arabs, Physical activity

## Abstract

**Background:**

Adequate sleep and physical activity have been linked to the overall well-being of both medical and psychiatric patients. Patients with schizophrenia have shown abnormal sleep patterns and decreased physical activity that were linked to their psychopathology and physical health. These phenomena are not studied yet in Arab patients with schizophrenia. The purpose of this study is to study the sleep and exercise patterns in Arab patients with schizophrenia compared with those of healthy controls.

**Method:**

A total of 99 patients with schizophrenia and 101 controls were recruited. Arabic versions of sleep, exercise, socio-demographic, and clinical questionnaires were administered as well as the validated scales to measure psychopathology, depression, and suicidality in these participants.

**Results:**

The majority of patients with schizophrenia slept more than 8 h per day and exercised less when compared with controls. Sleep quality was worse in those with higher depression score and higher suicidality scores were seen in patients with lower sleep duration. Multinomial regression showed that patients with schizophrenia have higher odds of sleeping more than 8 h even after controlling for the intake of antipsychotics, age, gender, smoking status, and other confounding factors.

**Conclusion:**

Our results showed that Arab patients with schizophrenia are at increased risk of having longer sleep duration with inadequate physical activity, which are correlating with worsening of depressive symptoms and suicidality. Thus, more attention should be paid to the changes in sleep patterns and level of exercise when treating Arab patients with schizophrenia.

**Electronic supplementary material:**

The online version of this article (10.1007/s12529-019-09830-2) contains supplementary material, which is available to authorized users.

## Introduction

Schizophrenia is a chronic mental disorder caused by a combination of genetic and environmental factors. The characteristic features of schizophrenia are: positive symptoms (hallucinations, delusions, and formal thought disorder), negative symptoms (emotional/social withdrawal, apathy, and lack of motivation) and general psychopathology (cognitive impairment, agitation, anxiety, and depression) [1]. The prevalence of schizophrenia worldwide is around 1% and patients with schizophrenia have higher morbidity and mortality rates when compared with the general population [1, 2]. Sleep disturbances occur in up to 80% of patients with schizophrenia and usually lead to impaired cognitive capacity and compromise the quality of life (QOL) [3, 4]. Polysomnography studies have shown changes in sleep architecture in schizophrenia patients compared with that in healthy controls, the former exhibiting longer sleep latency, reduced sleep efficiency, decreased deep sleep, and more night-time awakenings [5, 6].

Adequate sleep is crucial for optimal health, as it is during sleep that repair of bodily systems takes place; and the brain clears itself of metabolites and consolidates memory [7]. Research on sleep disturbances in newly admitted patients found that 83% of patients with acute schizophrenia had at least one type of sleep disorder (difficulty falling asleep, difficulty waking up, difficulty maintaining sleep, poor sleep quality, or increased time spent in bed). Sleep irregularities in schizophrenia patients lead to a deteriorating clinical condition. Patients who had a poor sleep exhibited lower scores on all QOL domains; they were more depressed and anxious with more adverse reactions to medications than those who had a good sleep [3]. Research also showed that sleep disturbances have been associated with increased levels of thought disorder and positive symptoms, and may foreshadow symptom relapse [4]. There is also data suggesting that poor sleep stimulates psychotic experiences, while enhanced sleep in individuals with psychosis may reduce their psychotic symptoms [7].

Due to its enormous benefits on health and sleep, exercise has been an appealing non-medical treatment for poor sleeping patterns [8]. Recent research, however, has indicated that poor sleep may impede the efforts of individuals to be physically active, highlighting the bidirectional association between sleep and exercise [9]. In their study, Faulkner & Sparkes [10] found that exercise can help decrease patients’ auditory hallucinations, boost self-regard, and improve sleeping patterns and overall behavior. The authors hypothesize that it might not be the exercise itself that provides these benefits, but rather the distraction and social interaction that they provide to patients [10]. Other studies also found that exercise significantly improved negative symptoms in patients with schizophrenia [11]. In that regard, physical activity has the prospect of enhancing the quality of life for schizophrenia patients through two means: physical and psychological [12]. Physically, schizophrenia patients are more prone to lead sedentary lifestyles than the general population [13] and are therefore at higher risk of having chronic illnesses associated with inactivity, such as obesity [14]; and exercise helps in reducing the latter.

The significance of sleep research in schizophrenia lies in the fact that sleep problems can be improved, and that sleep treatments have the potential to instigate significant improvements in clinical outcomes. Research shows that control of sleep-related symptoms regularly overlaps with control of clinical outcomes, indicating that treatment of sleep disturbances may be a useful target in enhancing clinical outcomes of schizophrenia patients [15]. Studies from the general populations have shown that individuals who slept 7–8 h reported fewer sleep complaints [16] and had lower mortality rates [17] than those who slept either < 7 or > 8 h. Earlier community studies also identified a positive association between a 7–8 h sleep duration and health status and longevity [18, 19]. Others found that on average, a 7.5-h sleeper from a working population has better outcomes in mental well-being, mood, and functional capacity [20]. Furthermore, a recent psychiatry commission on protection of physical health in patients with mental illness identified sleep disturbances and physical inactivity as two of the major modifiable factors that should be targeted to improve physical health of these patients [21].

Given the importance of the association between sleep and schizophrenia and the lack of research studying this relation in the Arab population, the objective of this project is to compare the sleeping patterns between Arab schizophrenia patients and healthy controls and to evaluate which factors predict sleep differences between these two groups. This study will also take into consideration the exercise patterns of the participants. Previous studies have shown that insomnia is common in never medicated patients and usually precedes further exacerbation of psychotic symptoms. However, medicated patients have better sleep efficiency with first generation antipsychotics (FGA) and longer sleep duration with second-generation antipsychotics (SGA) [6, 22]. As the majority of the schizophrenia patients are medicated, we hypothesized that patients with schizophrenia have longer sleep duration than healthy controls and that their mental illness itself is an independent predictor of irregular sleep duration, irrespective of other contributing variables. The research also showed a reciprocal relationship between sleep and exercise [23] and a recent meta-analysis of randomized controlled studies showed that exercise improves the sleep quality of patients with serious mental illness [24]. Thus, schizophrenia patients who have more sleep disturbances are expected to have poor exercise patterns.

## Methods

This study is a cross-sectional assessment of the self-reported sleep patterns in patients with schizophrenia. We conducted the study between February 2013 and November 2014. The Institutional Review Boards of Weill Cornell Medicine-Qatar and Hamad Medical Corporation (HMC, Doha, Qatar) approved the study. Participants recruited in our study signed a written informed consent after they received precise information about the procedures.

## Setting and Participants

Qatar is a fast-growing economy with a high number of expatriates from different nationalities residing in the state. Qataris and non-Qatari Arabs are the most stable populations in Qatar, and they represent 15% and 13% of the total population, respectively [25] (Qatar Statistic Authority 2010). We recruited patients with schizophrenia (*n* = 99) from the Psychiatry Hospital of HMC. The latter is the only psychiatric hospital in Qatar, comprising four inpatient units and ten outpatient clinics. The inpatient units accommodate up to 70 patients with a usual 90–95% occupancy rate, while the outpatient clinics receive approximately 120 patients per day, 25% of them carrying a schizophrenia diagnosis. The diagnosis of these patients was confirmed by the Arabic version of the Mini International Neuropsychiatric Interview (MINI-6) [26, 27], which relied on the Diagnostic and Statistical Manual of Mental Disorders (DSM-IV) criteria [28] (APA, 2000). The Arabic version of the MINI has been utilized before in several studies from Qatar [29–31]. Recruitment of the control group (*n* = 101) happened at the primary health care centers in Doha. The inclusion criteria for the schizophrenia group were as follows: (a) age between 18 and 65, (b) being an Arab (Qatari or non-Qatari), and (c) having a diagnosis of schizophrenia as per MINI-6 structured interview. The exclusion criteria were (a) drug or alcohol abuse during the last 6 months before recruitment into the study, (b) presence of DSM-IV diagnosis other than schizophrenia, and (c) risk of harming self or others. The inclusion and exclusion criteria for the control group were the same as above except that the participants should not have had any diagnosis of mental illness.

## Measures and Definitions

Participants answered a set of structured questionnaires that addressed the sociodemographic information, medical and psychiatric history, sleep patterns, and psychometric characteristics. Four raters were trained to administer these questionnaires and measures identically to all participants. We administered a sleep questionnaire, shown in the Electronic Supplementary Material 1 (ESM 1), to asses sleep patterns. A sleep score was devised based on this questionnaire. The score aimed to measure subjective sleep quality, sleep latency, sleep duration, sleep disturbances, and daytime dysfunction. Electronic Supplementary Material 2 (ESM 2) illustrates the calculation of component scores based on the questionnaire. Higher scores are indicative of the more inferior quality of sleep. The Arabic sleep questionnaire was derived from the Pittsburgh Sleep Quality Index (PSQI) [32] and was piloted in a small sample of Arab patients and controls before conducting the study. The feedback from the pilot study was incorporated in the final version used. However, there were no formal validation measures because of the lack of the proper Arabic gold standard to compare with. We calculated the total sleep duration by adding the number of hours slept at night and during day nap and was divided into three groups: (1) < 7 h referring to short sleep duration, (2) 7–8 h as the standard reference group, and (3) > 8 h as the long sleep duration. This grouping was chosen based on earlier studies that found a positive association between a 7–8 h sleep duration and health status and longevity [18, 19] and others who reported that on average, a 7.5 h sleeper has better outcomes in mental well-being, mood, and functional capacity [20]. We also measured the exercise activity of each subject during the month prior to assessment using the Arabic version (see Table 3).

Some researchers might mistrust the capacity of schizophrenia patients to report their sleep and exercise measures correctly, given the possible impairment of cognitive functioning associated with the illness. However, a study comparing self-reported sleep measures with polysomnography measures in schizophrenia patients established that they were highly correlated [33].

Further, clinical measures were used to quantify the psychopathology diagnosed. The Positive and Negative Syndrome Scale (PANSS) was used for disease severity assessment and comprised questions addressing positive symptoms, negative symptoms, and general observations in patients with schizophrenia. We used the Calgary Depression Scale for Schizophrenia (CDSS) to differentiate between depression and negative symptoms in schizophrenia patients and the International Suicide Prevention Trial (InterSePT) scale for the evaluation of suicidal ideation. All the scales used were the Arabic versions that were validated for use in Arab-speaking subjects [29, 34, 35]. Four research assistants were trained to administer these scales similarly to all subjects involved in the study. They established good inter-rater reliability on the different scales and questionnaires before obtaining the measures from the subjects involved in the study.

## Statistical Analysis

All statistical analyses were performed using the IBM-SPSS version 24 (IBM Corp, Armonk, NY, 2015), with statistical significance set at 0.05. Continuous variables were presented as the mean ± standard deviation (SD), while frequency distributions were used for categorical variables. For the assessment of different characteristics across the subject groups, participants were divided into two groups: schizophrenia patients and control participants. We compared the variables in these groups using chi-square tests to analyze categorical variables and independent sample *t* test for continuous variables. For the assessment of characteristics across the sleep duration groups, we divided the sample into three groups: < 7 h, 7–8 h, and > 8 h. Chi-square tests were used to compare categorical variables and one-way ANOVA to compare continuous ones. For correlations between continuous variables, we used Pearson correlation tests. All analyses were done with Bonferroni corrections for multiple comparisons. We also used the multinomial logistic regression to assess the factors that are contributing to variations in sleep duration. The significant variables from the above analyses and the clinically relevant ones from other studies were added as the independent predictors. The dependent variable in the regression equation was sleep duration, grouped into < 7 h, 7–8 h, and > 8 h. Goodness of fit was tested using the Nagelkerke Pseudo *R*^2^ test.

## Results

### Sample Characteristics by Subject Groups (Table 1)

The mean age was not significantly different between the schizophrenia and the control groups, and most of the subjects were between 25 and 44 years old. The following sociodemographic and clinical variables showed significant differences between the two groups: gender (χ^2^ = 11.703, *p* < .01), nationality (χ^2^ = 17.992, *p* < .01), marital status (χ^2^ = 52.884, *p* < .01), education (χ^2^ = 53.761, *p* < .01), and employment status (χ^2^ = 113.506, *p* < .01). Bonferroni-adjusted comparisons showed that there were significantly more males in the schizophrenia group and more females in the control group. The number of Qataris was significantly higher in the schizophrenia group than non-Qatari Arabs. The majority of the subjects in the schizophrenia group were single, unemployed with maximum intermediate schooling, while more subjects in the control group were married and employed with a graduate degree. There were also more smokers in the schizophrenia group than the control group (Table 1). There were no differences between the groups regarding medical history, i.e., having diabetes, hypertension, cholesterol, and high triglyceride levels (data not shown).

**Table 1:** Sociodemographic and medical characteristics of study participants

	Schizophrenia group (*n* = 99)	Control group (*n* = 101)
Age (mean ± SD)	34.84 ± 9.78	33.97 ± 8.30
Age groups *n* (%)		
≤ 24 years	12 (12.4%)	11 (10.9%)
25–34 years	37 (38.1%)	50 (49.5%)
35–44 years	30 (30.9%)	26 (25.7%)
≥ 45 years	18 (18.6%)	14 (13.9%)
Gender *n* (%)		
Male	66 (66.7%)^a^	43 (42.6%)
Female	33 (33.3%)	58 (57.4%)^b^
Nationality *n* (%)		
Qatari	64 (64.6%)^a^	35 (34.7%)
Arab (non-Qatari)	35 (35.4%)	66 (65.3%)^b^
Marital status *n* (%)		
Single	60 (61.2%)^a^	22 (21.8%)
Married Divorced/widowed	26 (26.5%) 12 (12.2%)^a^	78 (77.2%)^b^ 1 (1.0%)
Education *n* (%)		
No schooling	2 (2.0%)	0 (0.0%)
Elementary/intermediate	37 (37.4%)^a^	9 (8.9%)
Secondary	36 (36.4%)^a^	16 (15.8%)
Vocational/college/graduate	24 (24.2%)	76 (75.2%)^b^
Employment *n* (%)		
Employed	26 (26.3%)	100 (99.0%)^b^
Unemployed	73 (73.7%)^a^	1 (1.0%)
Smoker *n* (%)		
Yes	62 (62.6%)^a^	31 (30.7%)

^a^higher than the control group

^b^higher than the schizophrenia group (*p* < .05)

### Sleep Patterns by Subject Groups (Table 2)

Table 2 presents the responses to the sleep questionnaire (ESM 1) for each subject group. The factors related to duration of sleep (*F*_1,197_ = 16.167, *p* < .01), time of sleep (χ^2^ = 17.811, *p* < .01), and time of waking up (χ^2^ = 44.802, *p* < .01) showed significant differences between the two groups. The mean duration of sleep (with or without nap time) was significantly higher in the schizophrenia group than the control group. There was no significant difference in the number of subjects taking naps between the two groups. The number of patients sleeping > 8 h in 24 h was also significantly higher in the schizophrenia group, and more schizophrenia subjects slept earlier and woke up later than healthy subjects (Table 2). There were no significant differences in the subjects’ rating of sleep quality, early or late insomnia, problems during sleep or tiredness during the day (Table 2). The sleep score, calculated as per ESM 2, was higher in the schizophrenia group than the control group, but this difference did not reach statistical significance (Table 2).

**Table 2:** Sleeping patterns of study participants

	Schizophrenia group (n = 99)	Control group (n = 101)
Sleep time in 24 h (mean ± SD)	9.12 ± 2.27^a^	7.99 ± 1.65
Night sleep time (mean ± SD)	8.21 ± 1.91^a^	6.81 ± 1.12
Taking naps *n* (%)		
Yes	47 (47.5%)	56 (55.45%)
No	52 (52.53%)	45 (44.55%)
Groups of hours slept *n* (%)		
7–8 h	32 (32.7%)	45 (45.0%)
< 7 h > 8 h	11 (11.2%) 55 (56.1%)^a^	20 (20.0%) 35 (35.0%)
Time of sleep *n* (%)		
Between 7 and 9 pm	11 (11.1%)^a^	2 (2.0%)
Between 9 and 11 pm	33 (33.3%)^a^	19 (18.8%)
Between 11 pm and 1 am	38 (38.4%)	66 (65.3%)^b^
Between 1 and 3 pm	11 (11.1%)	9 (8.9%)
After 3 am	6 (6.1%)	5 (5.0%)
Time of wakeup *n* (%)		
Between 2 and 4 am	7 (7.1%)	2 (2.0%)
Between 4 and 6 am	24 (24.2%)	69 (69.0%)^b^
Between 6 and 8 am	35 (35.4%)^a^	22 (22.0%)
Between 8 and 10 am	15 (15.2%)^a^	2 (2.0%)
After 10 am	18 (18.2%)^a^	5 (5.0%)
Difficulty falling asleep *n* (%)	32 (32.7%)	22 (21.8%)
Early wakeup *n* (%)	33 (33.3%)	29 (28.7%)
Difficulty falling back into sleep *n (%)*	29 (29.9%)	21 (20.8%)
Tiredness/sleepiness *n* (%)	35 (35.7%)	46 (45.5%)
Problems during sleep *n* (%)	36 (36.4%)	30 (29.7%)
Participant rating of sleep quality *n* (%)		
1	4 (4.0%)	3 (3.0%)
2	9 (9.1%)	4 (4.0%)
3	23 (23.2%)	34 (33.7%)
4	29 (29.3%)	33 (32.7%)
5	34 (34.3%)	27 (26.7%)
Sleep score (mean ± SD)	3.56 ± 2.47	3.25 ± 2.07

^a^higher than the control group

^b^higher than the schizophrenia group (*p* < .05)

### Exercise Patterns by Subject Groups (Table 3)

Table 3 presents the responses to the exercise questionnaire for each subject group. The factors related to frequency (χ^2^ = 15.469, *p* = .001) and intensity of exercise (χ^2^ = 6.839, *p* = .033) showed significant differences between the two groups. The mean frequency of exercise was significantly higher in the control group than in the schizophrenia group. Among those who exercised, more schizophrenia patients did light exercise, while more control subjects practiced moderate intensity exercise (Table 3). There were no significant differences in the duration or reason for exercising.

**Table 3:** Exercise patterns of study participants

	Schizophrenia group (*n* = 99)	Control group (*n* = 101)
Frequency of exercise *n* (%)		
Once per week	6 (6.1%)	20 (19.8%)^b^
2–4 times per week	11 (11.1%)	22 (21.8%)^b^
> 5 times per week	14 (14.1%)	13 (12.9%)
Never	68 (68.7%)^a^	46 (45.5%)
Duration of exercise *n* (%)		
< 15 min	7 (23.3%)	6 (11.3%)
15–30 min	12 (40.0%)	19 (35.8%)
30–60 min	3 (10.0%)	17 (32.1%)
> 1 h	8 (26.7%)	11 (20.8%)
Intensity of exercise *n* (%)		
Light	26 (86.7%)^a^	33 (61.1%)
Moderate	1 (3.3%)	12 (22.2%)^b^
High	3 (10.0%)	9 (16.7%)
Reason for exercise *n* (%)		
Reducing/maintaining weight	9 (29.0%)	17 (32.1%)
General health	16 (51.6%)	20 (37.7%)
Pleasure/leisure	3 (9.7%)	7 (13.2%)
Stress relief	2 (6.5%)	2 (3.8%)
Other	1 (3.2%)	7 (13.2%)

^a^higher than the control group

^b^higher than the schizophrenia group (*p* < .05)

### Psychiatric Profile of Schizophrenia Group (Table 4)

Table 4 displays the psychiatric characteristics of patients in the schizophrenia group. The mean duration of illness was over 11 years and about one-third were not taking their medications at the time of assessment. The majority of patients were on SGA. About one-third also had history of suicidal attempt and aggressive behavior. The mean scores on PANSS and CDSS (Table 4) reflected moderate psychopathology. We also ran a Pearson correlation test between each of these variables with the measures on sleep duration and sleep score. InterSept and CDSS scores were significantly correlated with sleep duration and sleep quality, respectively. Higher InterSept scores showed significant correlation with lower sleep duration (Pearson correlation = − .258, *p* = .013) and higher CDSS scores with higher sleep scores (i.e., poorer sleep quality) (Pearson correlation = .294, *p* = .005).

**Table 4:** Psychiatric profile of schizophrenia group

	Schizophrenia group (*n* = 99)
Duration of illness (mean ± SD)	11.72 ± 8.70
Age at onset of psychiatric symptoms (mean ± SD)	22.89 ± 8.55
Antipsychotic use	
On antipsychotics	69.7%
Not on antipsychotics	30.3%
Age at administration of first psychotropic medication (mean ± SD)	24.69 ± 8.65
Number of psychiatric hospitalizations (mean ± SD)	3.91 ± 2.90
History of suicide attempts *n* (%)	28 (30.8%)
Number of suicide attempts (mean ± SD)	2.24 ± 1.67
History of aggressive behavior *n* (%)	35 (37.6%)
Antipsychotics *n* (%)	
None	30 (30.3%)
FGA	8 (8.1%)
SGA	44 (44.4%)
Both FGA & SGA	17 (17.2%)
PANSS Total Score (mean ± SD)	74.15 ± 23.38
InterSePT Total Score (mean ± SD)	3.02 ± 4.68
CDSS Total Score (mean ± SD)	4.14 ± 4.13

*FGA*, First generation antipsychotics, *SGA*, Second generation antipsychotics, *PANSS*, Positive and Negative Syndrome Scale, *InterSePT*, International Suicide Prevention Trial Scale, *CDSS*, Calgary Depression Scale for Schizophrenia

### Sample Characteristics by Sleep Duration Groups (Table 5)

In order to analyze the associations between the psychiatric measures and sleep duration, we compared the sample variables among the three sleep duration groups. Our results showed that diagnosis (χ^2^ = 9.233, *p* = .01), employment status (χ^2^ = 8.466, *p* = .015) and smoking (χ^2^ = 7.059, *p* = .029) were statistically significant (Table 5). Post hoc comparisons showed that subjects who slept > 8 h were mostly schizophrenia patients, while the majority of the control subjects slept < 7 h or 7–8 h*.* There were also significantly more unemployed people and more smokers in the group with > 8 h of sleep when compared with the 7–8 h group (Table 5). Marital status or level of education showed no significant difference between the sleep duration groups.

**Table 5:** Sociodemographic and medical characteristics of participants by sleep duration groups

	7–8 h (*n* = 77)	< 7 h (*n* = 31)	8 h (*n* = 90)
Age (years; mean ± SD)	35.32 ± 9.46	35.47 ± 9.07	33.36 ± 8.70
Age groups *n* (%)			
≤ 24 years	9 (11.7%)	2 (6.7%)	12 (13.5%)
25–34 years	28 (36.4%)	15 (50.0%)	42 (47.2%)
35–44 years	27 (35.1%)	8 (26.7%)	21 (23.6%)
≥ 45 years	13 (16.9%)	5 (16.7%)	14 (15.7%)
Gender *n* (%)			
Male	39 (50.6%)	16 (51.6%)	53 (58.9%)
Female	38 (49.4%)	15 (48.4%)	37 (41.1%)
Participant grouping *n* (%)			
Schizophrenia	32 (41.6%)	11 (35.5%)	55 (61.1%)^a,b^
Control	45 (58.4%)^c^	20 (64.5%)^c^	35 (38.9%)
Nationality *n* (%)			
Qatari	37 (48.1%)	12 (38.7%)	49 (54.4%)
Arab (non-Qatari)	40 (51.9%)	19 (61.3%)	41 (45.6%)
Marital status *n* (%)			
Single	31 (40.3%)	10 (32.3%)	40 (44.9%)
Married	41 (53.2%)	17 (54.8%)	46 (51.7%)
Divorced/widowed	5 (6.5%)	4 (12.9%)	3 (3.4%)
Education *n* (%)			
No schooling	0 (0.0%)	1 (3.2%)	1 (1.1%)
Elementary/intermediate	15 (19.5%)	7 (22.6%)	23 (25.6%)
Secondary	24 (31.2%)	3 (9.7%)	25 (27.8%)
Vocational/college/graduate	38 (49.4%)	20 (64.5%)	41 (45.6%)
Employment *n* (%)			
Employed	56 (72.7%)^c^	22 (71.0%)	47 (52.2%)
Unemployed	21 (27.3%)	9 (29.0%)	43 (47.8%)^a^
Smoker *n* (%)			
Yes	27 (35.1%)	15 (48.4%)	50 (55.6%)^a^

^a^higher than the 7–8 h

^b^higher than the < 7 h group

^c^higher than the > 8 h group (*p* < 0.05)

### Exercise Characteristics by Sleep Duration Groups

There were no significant differences in exercise patterns when they were compared across sleep duration groups (data not displayed).

### Psychiatric Characteristics by Sleep Duration Groups (Table 6)

Table 6 displays the psychiatric characteristics of schizophrenia patients when compared across sleep duration groups. The InterSept total score was significantly higher in patients who slept < 7 h than those who slept > 8 h (*F*_2,92_ = 4.127, *p* = .019).

**Table 6:** Psychiatric characteristics of schizophrenia patients by sleep duration groups

	7–8 h (*n* = 32)	< 7 h (*n* = 11)	> 8 h (*n* = 55)
Duration of illness (mean ± SD)	12.59 ± 8.55	11.00 ± 11.55	11.38 ± 8.60
Age at onset of psychiatric symptoms (mean ± SD)	22.04 ± 8.49	27.63 ± 6.99	22.65 ± 8.80
Age at administration of first psychotropic medication (mean ± SD)	24.00 ± 8.79	27.50 ± 6.70	24.67 ± 8.99
Number of psychiatric hospitalizations (mean ± SD)	3.35 ± 2.00	2.29 ± 1.25	4.51 ± 3.34
History of suicide attempts *n* (%)	10 (33.3%)	5 (55.6%)	12 (23.5%)
Number of suicide attempts (mean ± SD)	1.75 ± 0.71	2.60 ± 2.61	2.55 ± 1.75
Antipsychotics *n* (%)			
Not on antipsychotics	10 (31.3%)	2 (18.2%)	18 (32.7%)
On antipsychotics	22 (58.8%)	9 (81.8%)	37 (67.3%)
PANSS Total Score (mean ± SD)	70.66 ± 19.61	79.80 ± 23.12	75.17 ± 25.49
InterSePT Total Score (mean ± SD)	3.06 ± 5.38	7.00 ± 4.69^a^	2.31 ± 3.88
CDSS Total Score (mean ± SD)	3.90 ± 3.73	4.00 ± 4.34	4.38 ± 4.38

*PANSS*, Positive and Negative Syndrome Scale, *InterSePT*, International Suicide Prevention Trial Scale, *CDSS*, Calgary Depression Scale for Schizophrenia

^a^higher than the > 8 h group (*p* < .05)

### Multinomial Logistic Regression and Sleep Duration (Table 7)

Using the variables that were significant (and clinically relevant) in the previous analyses, a forward stepwise multinomial logistic regression was carried out to examine what factors were independent contributors to sleep duration. The 7–8 h was the reference category with which to compare each of the other two groups (< 7 h or > 8 h). The predictors entered into the regression model were the diagnosis, age, gender, smoking, antipsychotic use, PANSS, InterSept, and CDSS total scores. The full regression model with all the predictors was fitting (*p* = .002) and significant (χ2 = 348.475, df = 354). The Nagelkerke *R*^2^ value was 0.104. The predictors that remained significant after controlling for the other independent contributors were diagnosis and InterSept score for the group who slept < 7 h, and diagnosis for the group who slept > 8 h, as compared with the 7-8 h group (Table 7). In the < 7 h group, healthy controls were at 3.48 times higher odds of having a shorter sleep duration than schizophrenia patients. Additionally, participants with higher InterSept scores were at higher risk of shorter sleep duration than those with lower suicidality scores. In the > 8 h group, schizophrenia patients had 2.052 times higher odds of having longer sleep duration than their control counterparts.

**Table 7:** Predictors of sleep duration using multinomial logistic regression

Sleep duration	Independent contributors	Exp (B)	95% CI for Exp (B)		*p* value
			Lower bound	Upper bound	
< 7 h	Diagnosis				
	Schizophrenia Control	0.287 -	0.089 -	0.929 -	.037
	InterSePT score	1.175	1.032	1.337	.015
> 8 h	Diagnosis				
	Schizophrenia Control	2.052 -	1.040 -	4.026 -	.037

Reference category is the group sleeping 7–8 h. *InterSePT*, International Suicide Prevention Trial Scale

## Discussion

Our study examined the differences in self-reported sleep and exercise patterns between patients with schizophrenia and healthy controls. We also studied the relationship between sleep duration and the psychopathological measures of our participants. The results will be discussed below according to these objectives.

The schizophrenia group was mostly males, Qataris, and smokers. Males are usually admitted more than females as they have a worse prognosis [36, 37]. Although Qataris are not the majority in the population residing in Qatar, they are indeed the most stable; expatriates with unstable schizophrenia who are unable to work would end up leaving Qatar to their country of origin. Smoking is a common comorbidity in patients with schizophrenia [38], where about 75% of patients with schizophrenia smoke more than two packs of cigarettes per day [14]. Earlier studies also showed that smoking was associated with difficulty initiating sleep and difficulty waking up [39]. Additionally, the nicotine present in cigarettes stimulates neurotransmitters involved in the circadian cycle, thereby affecting the duration and quality of sleep [40].

Our results also indicated that there was an association between the diagnosis of schizophrenia and longer sleep duration. The majority of patients were sleeping > 8 h, and they were sleeping earlier and waking up later than the control group. The sleep quality was worse in patients with schizophrenia, but this difference did not reach statistical significance. Research has shown that shorter sleep duration was more common in non-medicated schizophrenia patients, while medicated patients showed longer sleep duration [41]. This increased sleep duration in medicated schizophrenia patients was attributed to increased sleep time, both during the night and during day-time naps [42]. A similar study by Martin et al. (2005) showed that, compared with healthy controls, schizophrenia patients reported later wake-up times in the morning, more time spent in bed, a higher frequency of daytime naps, and an increased total sleep duration [43, 44]. Research also points to disruptions in the internal circadian clock of patients, showing abnormal melatonin levels [41] that might also be involved in irregular sleep durations. In his study on that matter, Yates (2016) suggested that sleep disturbances might cause an increase in dopamine levels in the brain, which in turn increases vulnerability to psychosis; further, increased dopamine might elevate the risk of sleep disturbances, creating as such a positive feedback loop where sleep and dopamine irregularities intensify each other [15]. A systematic review of sleep in schizophrenia also supported the finding of the dopamine system dysfunction in the sleep-wake cycle [6]. Melatonin is known to control this loop, but there is evidence that schizophrenia patients have abnormal melatonin control, hence allowing for increased sleep irregularities [15]. Treatment with antipsychotics are known to alter sleep patterns, but our regression model has shown that even after controlling for antipsychotic use, the schizophrenia diagnosis alone was found to be an independent contributor to longer sleep duration. Some studies implicated cytokines as the mediator for the effects of AP on sleep patterns [45, 46].

Schizophrenia patients in our sample exercised less frequently and with lighter intensity than healthy subjects. This finding is in line with other studies that demonstrated that patients with schizophrenia had lower levels of physical activity than the general population [47]. Research also indicates that this inactivity contributes to illnesses like cardiac disease, diabetes, obesity, and metabolic syndrome [48], and hence targeting physical activity in schizophrenia patients can help enhance their physical health and reduce morbidity rates among them. Further, physical activity has been associated with better symptomatology in schizophrenia patients, leading to a better sleep [10, 11]. A recent study reported that sleep quality correlated positively with duration of moderate physical activity per week [49].

Our results also showed that in the schizophrenia group higher InterSept scores were associated with shorter sleep duration, and higher CDSS scores were associated with worsening of sleep quality. In other words, abnormal sleep duration and quality were associated with an increased risk of suicidality and depression. Our regression also showed that shorter sleep duration was independently associated with higher InterSept scores. These findings are in line with other studies in the literature, where sleep disturbances were linked to increased depressive symptoms and suicidality in patients with schizophrenia [50]. Suicidal behaviors were also reported to increase in patients with sleep disturbance and mental illness in general [51, 52]. Some studies found that depressive symptoms were associated with more inferior sleep quality in adolescents [53], while others studied sleep quality in postpartum women and also found that it was significantly associated with worse symptoms of depression and anxiety [54]. Further, insomnia and daytime somnolence were also associated with more psychotic-like experiences in adolescents [55].

Other studies have also established that people with short sleep duration were more likely to have suicidal ideation than those whose sleep duration was 7 h [56, 57]. A study by Lee et al. (2012) [55] also found that insufficient sleep duration increases suicidality independently of self-reported depressive symptoms. Adequate sleep duration is essential in the regulation of physical functions and the overall well-being of the human body as it enables functional recovery of central nerves that are wearied out during the day; as such, insufficient sleep worsens physical and psychological functions [56]. Further, some other variables could make individuals with short sleep duration more susceptible to suicidality, e.g., low impulse control, which is common in patients with schizophrenia, has been described to be correlated with short sleep duration [55].

There are no formal studies on the sleep and exercise health styles in Arab patients with mental illness in Qatar. Qualitative and quantitative studies from the general population in Qatar and other Arab countries pointed to several cultural and social factors that are contributing to the lack of adequate exercise and sleep hygiene in Arab populations. These studies showed that the majority are aware of the importance of exercise and sleep to their wellbeing but also identified factors that hinder their compliance with appropriate exercise and sleep habits [58, 59]. Among these are (1) the hot weather in Qatar that prevents people from exercising outside; (2) staying up late at night with the common practice of day naps or “siesta” where the work schedule allows people to have long break during the day and thus increase the sleep hours; (3) smoking cigarettes and “nargila” are common practices in these populations especially among patients with mental illness; and (4) gender and age differences where mostly females and older people, in general, feel “shame” of exercising in public places. These matters and others should be discussed with patients and proper interventions at the individual, family, and societal levels should be implemented to improve the physical and mental health of the population in general and in particular for patients with serious mental illness.

### Limitations

The study has several strengths like the presence of a control group that was assessed by the same raters and instruments. However, there are a few limitations that might affect the generalizability of the results. First, our sample size is not ideal; a larger sample size would have given more power to our findings and allowed for more accurate identification of variables that are associated with sleep and exercise in schizophrenia patients. Second, the cross-sectional design of this study limits the conclusion of a causative effect between sleep and schizophrenia. We found an association between the two, but it is unknown which causes the other, or if it is a positive feedback cycle where both factors feed into each other. Third, although the self-reported measures have been shown to be reliable, the more objective ones using polysomnography or actigraphy are better to cover all the necessary measures of sleep duration and quality. Fourth, the Arabic sleep questionnaire is developed locally and was not formally validated, which could compromise the comparisons with other results obtained from standardized instruments. Further, even though our results did not show antipsychotic use as an independent predictor, the latter might be increasing sleep duration and augmenting the positive association we found between sleep duration and schizophrenia diagnosis. Thus, more prospective studies would allow a better understanding of the complex relationships between sleep, schizophrenia, and antipsychotic use.

## Conclusions

To the best of our knowledge, this is the first study in the Arab population to assess the relationships between sleep and schizophrenia. There are significant changes in the sleep patterns in Arab patients with schizophrenia that can impact the clinical outcomes. In conclusion, sleep hygiene and monitoring patients before and after treatment for any pathological changes in their sleep patterns might avoid the adverse effects on sleep and thus improve the overall wellbeing.

### Electronic Supplementary Material

ESM 1 (DOCX 16 kb)

ESM 2 (DOCX 15 kb)
