# Correction to: Self-Reported Sleep and Exercise Patterns in Patients with Schizophrenia: A Cross-Sectional Comparative Study

**DOI:** 10.1007/s12529-020-09851-2

**Published:** 2020-01-22

**Authors:** Nancy Kiwan, Ziyad Mahfoud, Suhaila Ghuloum, Rifka Chamali, Arij Yehya, Samer Hammoudeh, Yahya Hani, Iman Amro, Hassen Al-Amin

**Affiliations:** 1 Department of Research, Weill Cornell Medicine-Qatar, Doha, Qatar; 2 Department of Health Policy and Research, Weill Cornell Medicine-Qatar, Doha, Qatar; 3 Department of Healthcare Policy and Research, Weill Cornell Medicine, New York, NY USA; 4 Psychiatry Hospital, Hamad Medical Corporation, Doha, Qatar; 5 Department of Psychiatry, Weill Cornell Medicine-Qatar, Education City, P.O. Box 24144, Doha, Qatar

**Correction to: International Journal of Behavioral Medicine**

**https://doi.org/10.1007/s12529-019-09830-2**

The original article has been corrected:

The article Self-Reported Sleep and Exercise Patterns in Patients with Schizophrenia: a Cross-Sectional Comparative Study written by Nancy Kiwan, Ziyad Mahfoud, Suhaila Ghuloum, Rifka Chamali, Arij Yehya, Samer Hammoudeh, Yahya Hani, Iman Amro, and Hassen Al-Amin was originally published electronically on the publisher’s internet portal (currently SpringerLink) on 17 December 2019 incorrectly without Open Access.

This is now corrected, and the copyright of the article changed on January 2020 to © The Author(s) 2020 and the article is forthwith distributed under the terms of the Creative Commons Attribution 4.0 International License (https://creativecommons.org/licenses/by/4.0/), which permits use, duplication, adaptation, distribution, and reproduction in any medium or format, as long as appropriate credit is given to the original author(s) and the source, a link to the Creative Commons license is provided, and any changes that were made are specified.

