# Supplementary material for: Self-Reported Sleep and Exercise Patterns in Patients with Schizophrenia: a Cross-Sectional Comparative Study
Source: Int J Behav Med. 2019 Dec 17;27(4):366–77. doi: 10.1007/s12529-019-09830-2 (PMC7359133; doi:10.1007/s12529-019-09830-2)
Supplement: Supplementary file 1 — (DOCX 16 kb) [file 12529_2019_9830_MOESM1_ESM.docx]

**ESM 1**  Sleep Questionnaire

| Questions | |
| --- | --- |
| 1 | How many hours do you, on average, sleep per day? |
| 2 | At what time do you usually go to bed? |
| 3 | At what time do you usually wake up? |
| 4 | Do you usually take naps during the day? |
| 5 | Approximately how long is your nap? |
| 6 | Do you often have difficulties falling asleep? |
| 7 | Do you wake up earlier than you want? |
| 8 | Do you often wake up after a short sleep and then have difficulty falling asleep again? |
| 9 | Do you often feel tired/ sleepy during the day? |
| 10 | Has anybody told you that you have any problems during sleep, like snoring, difficulty breathing, sleepwalking or others? |
| 11 | What kind of problems during sleep do you have? |
| 12 | How do you grade your sleep quality over the last six months? |
