# Supplementary material for: Self-Reported Sleep and Exercise Patterns in Patients with Schizophrenia: a Cross-Sectional Comparative Study
Source: Int J Behav Med. 2019 Dec 17;27(4):366–77. doi: 10.1007/s12529-019-09830-2 (PMC7359133; doi:10.1007/s12529-019-09830-2)
Supplement: Supplementary file 2 — (DOCX 15 kb) [file 12529_2019_9830_MOESM2_ESM.docx]

**ESM 2** Calculation of Sleep Score

| Component | Questions | Response | Score |
| --- | --- | --- | --- |
| 1. Subjective sleep quality (C1) | 12  (How would you rate the quality of your sleep over the last six months? 1= Poor, 5=Excellent) | 1  2  3  4  5 | 4  3  2  1  0 |
| 2. Sleep latency (C2) | 6  (Do you often have difficulties falling asleep?) | Yes  No | 1  0 |
| 3. Sleep duration (C3) | 1 & 5  (How many hours do you on average sleep per day? Approximately how long is your nap?) | Add number of hours slept at night and nap duration  <7  7-8  >8 | 1  0  1 |
| 4. Sleep disturbances (C4) | 7, 8 & 10  (Do you wake up earlier than you want? Do you often wake up after a short sleep and then have a difficulty falling asleep again? Has anybody told you that you have problems during sleep, like snoring, difficulty breathing, sleepwalking or others?) | Q7, Q8 & Q10:  Yes  No | 1  0 |
|  |  | Sum of Q7, Q8 & Q10 scores:  0  1  2  3 | 0  1  2  3 |
| 5.Daytime dysfunction (C5) | 9  (Do you often feel tired/ sleepy during the day?) | Yes  No | 1  0 |
| Total Sleep Score= C1+C1+C3+C4+C5 | | | |
